# Read-Right: a “web app” that improves reading speeds in patients with hemianopia

**DOI:** 10.1007/s00415-012-6549-8

**Published:** 2012-06-12

**Authors:** Yean-Hoon Ong, Maurice M. Brown, Patrick Robinson, Gordon T. Plant, Masud Husain, Alexander P. Leff

**Affiliations:** 1 UCL Multimedia, Information Services Division, University College, London, UK; 2 The National Hospital for Neurology and Neurosurgery, Queen Square, London, UK; 3 Institute of Cognitive Neuroscience, 17 Queen Square, London, WC1N 3AR UK; 4 The Department of Brain Repair and Rehabilitation, Institute of Neurology, University College London, London, UK

**Keywords:** Hemianopia, Alexia, Stroke, Internet, Rehabilitation

## Abstract

Effective behavioral therapies exist for patients with brain injury. The main issue is one of access. Can the internet be used as a resource so that suitable patients can build up enough practice to improve? We tested this hypothesis using a web-based application for patients with a right-sided hemianopia causing slow text reading. We studied 33 patients aged 26–81 years who fitted the entry criteria and accessed the therapy website between May 2010 and December 2011, in a longitudinal cohort study. The therapy consisted of reading animated, laterally scrolling text whose content and form was selected by the patients. Reading speeds on static text (main outcome) were assessed after every 5-h period of practice had been accrued. Statistical analysis was carried out using a repeated measures ANOVA. Read-Right therapy produced significant improvements in text reading speeds at all time points with a clear dose effect: 10 % at 5 h, 20 % at 10 h, 39 % at 15 h and 46 % at 20 h. Sub-analyses demonstrated that this was unlikely to be due to either multiple exposure to the testing materials (familiarity) or to the simple passage of time. This is the first example of a clinically proven therapy being delivered effectively to stroke patients over the internet. As therapists’ time is more limited than patients’ capacity to improve, carefully designed, web-based resources like Read-Right represent a realistic way of delivering a sufficient therapy dose to patients so they can obtain clinically meaningful improvements.

## Introduction

Persistent hemianopia occurs in approximately 20 % of people with stroke, the other major causes being head injury and tumors [3]. Hemianopia rarely improves beyond 6 months from the onset, so many patients are left with a fixed deficit [11]. Hemianopia has an adverse effect on activities of daily living, with reading affected in 80 % [10]. Despite this seemingly gloomy situation, hemianopic patients can continue to make functional improvements over time because other brain functions, such as those involved in controlling eye movements, can be engaged by strategy-based therapies, such as those that retrain eye movements, to partially compensate for their visual loss [7].

There are several different eye movement based therapies that have been shown to improve reading speeds [13]; one of the most popular methods uses animated, laterally scrolling text [8]. When this type of text is viewed, it induces an involuntary eye movement called small-field optokinetic nystagmus (see: http://www.readright.ucl.ac.uk/help/h_vid_eye.php). Crucially, this therapy induces an involuntary saccade into the patient’s blind field. Sufficient practice with this improves patients’ rightward reading eye movements and text reading speed when they return to reading normal, static text [9].

We wished to make this therapy freely available for patients to access and also to establish whether the therapy is effective outside the confines of a clinical trial. To do this we developed Read-Right (http://www.readright.ucl.ac.uk). This site provides diagnostic and therapeutic tools that patients, carers and medical staff can access from anywhere in the world.

## Methods

### Subjects

This study was approved by the UCL Research Ethics Committee. The patients in this study were self-selected. They registered with a valid email address then logged into the therapy website to download the Read-Right application, signing an online consent form in the process. Before having access to the therapy (laterally scrolling text, see below) they had to carry out baseline assessments of their visual fields and text reading speed. Inclusion criteria for the analysis were the following: (1) to have completed more than 5 h of therapy, and (2) to have a fixed right visual field homonymous deficit as defined by one or more missed stimuli on the automated visual field test, and (3) to have a baseline text reading speed greater than 40 words per minute (no upper limit was used as all patients were at least one standard deviation below the mean normal reading speed). The lower limit was used to try and exclude subjects with ‘pure’ alexia. A total of 43 patients met the first 2 criteria but 10 failed the third (text reading too slow), resulting in 33 patients. Two-thirds were male with an average age of 62 years. Median time since stroke, in those for whom we could ascertain this, was 15.7 months (interquartile range 2.6–59.6 months) (see Table 1).

**Table 1:** Demographic details for the 33 patients

Patient	Sex	Age	Cause (I,H, AVM)	Time since stroke (months)	Reading speed (wpm)	Change (%) in reading speed: 5 h	Change (%) in reading speed: 10 h	Extent of field defect	Post therapy rating	Reading more?
1	M	80			45	44		1		
2	M	48	I	58.6	106	1	36	1	7	3
3	F	26			129	−3		1		
4	M	48	I	34.2	64	27	75	2.5	10	3
5	M	78	I	3.5	145	4	−2	2.5	8	2
6	M	59	I	2.9	174	22	68	1	8	3
7	M	64			57	27	40	1		
8	M	73			100	10	47	1		
9	M	65			65	37	40	2.5		
10	F	81			60	50	57	10		
11	M	70	I	2.2	208	−10	−29	5	7	0
12	M	80	I	4.2	170	32	36	5	5	3
13	F	49			44	47	84	1		
14	F	60	H	217.5	128	23	13	2.5	9	0
15	F	57			44	22	17	2.5		
16	M	62	I	30.4	54	−18		1	3	1
17	M	52			109	23		1		
18	M	55			44	38		1		
19	F	43			78	−48	−46	1		
20	M	50	I	15.7	101	−9		1	4	3
21	M	65			152	−1	12	10		
22	M	74	I	0.4	66	56	56	2.5	8	3
23	F	56	H	100.9	61	−27	82	2.5	10	3
24	M	75	I	60.6	63	−25	2	1	8	3
25	F	77	I	0.3	78	4	3	1	7	3
26	F	57	I	18.7	102	4	−17	1	8	0
27	M	81			54	25	23	1		
28	F	75			75	43	−29	5		
29	M	45	H	842.2	61	23	7	1	10	3
30	M	66	I	1	189	3	16	2.5	7	1
31	F	37	AVM	7.5	70	19	11	2.5	10	2
32	M	67	I	1.4	125	7	10	1	10	3
33	M	78			109	−7	−12	2.5		
	M2:F1	62.2		77.9	94.6	10	20		7.7	2.2

*I* infarct, *H* hemorrhage, *AVM* arterio-venous malformation, *WPM* words/min, Reading more: *0* not reading more, *1* 15 min a day more, *2* 30 min a day more, *3* 1 h or more a day

### Automated visual field test

**Fig. 1:**
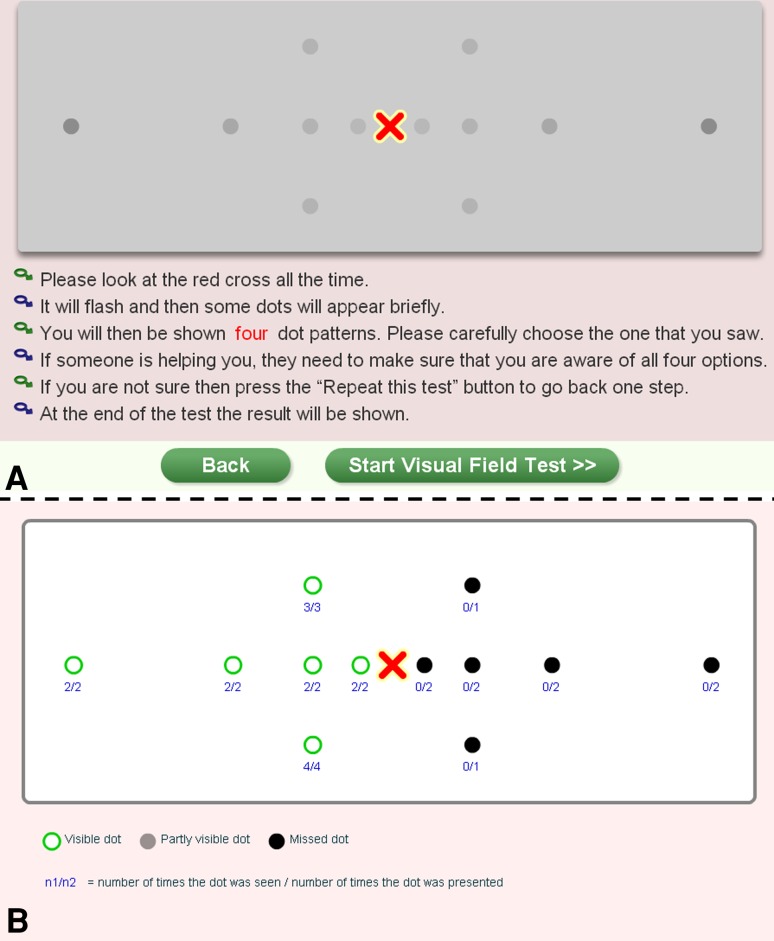
Screenshot of the visual field test (*top* **a**). Note the increasing contrast between dots (0.5° diameter) and background with increasing eccentricity. Below (**b**), results from a participant with a right-side, homonymous hemianopia

We developed a novel, adaptive, automated visual field test for assessing hemianopia in patients with text reading difficulties. We tested six points at 1°, 2.5°, 5° and 10° eccentricity from the fixation cross in both visual fields; four in each along the horizontal meridian, as this is key for text reading (Fig. 1; see: http://www.readright.ucl.ac.uk/help/h_vft.php). This test has been validated by comparing it with a clinical ‘gold standard’, the Humphrey automated perimeter (both 10–2 and 24–2 protocols), and has sensitivities in the range of 0.8–1 and specificities of 0.75–1 for the affected hemifield along the horizontal meridian [5]. In most subjects (26/33) we were able to obtain pre- and post-therapy fields to see if these changed over time.

### Text reading test

In order to evaluate the effect of the Read-Right therapy we developed a novel, online, timed reading test. Test materials consist of six standardized paragraphs of edited newspaper text 49 words in length, spread over seven lines. Patients initiated a countdown timer and then read the whole of the text, signaling when they had finished with a button press, at which point the timer recorded their reading speed. Each text was followed immediately by a short yes/no question, which varied and was related to the passage just read, to encourage patients to read the whole of the text. At each point in time subjects read three texts (a triplet); times were averaged to produce their reading speed. We averaged two measurements at baseline (two triplets containing all six texts between them) to improve precision. The patients correctly answered the comprehension question with an average 91.5 % accuracy. Incorrect trials were not excluded from the analysis.

The 6 texts were garnered from 13 examples read by 114 volunteers using the website. We chose the six with the least within- and between-subject variance. A total of 38 age-matched controls from the pool of 114 (mean = 59.7 years) read the six texts at an average speed of 302 wpm (words per minute; SD = 80). The texts were split into two halves (median split: easier three and harder three) and each triplet consisted of a mix of texts from each side of the median split. The order of presentation of the triplets was pseudo-randomized both within and across subjects according to the following rules: for each consecutive pair of points in time, all six texts were used; two easy or two hard texts were included in each triplet; no two adjacent tests had more than one text in common. This lead to 38 triplet types which were combined in a Latin square design across subjects.

### Therapy

The therapy consisted of reading laterally scrolling text (from right-to-left). Patients could control the speed, color (background and foreground), and content of what they read, choosing from a library of books and ever-changing really simple syndication (RSS) text feeds from the BBC website; (see, http://www.readright.ucl.ac.uk/help/h_vid_therapy.php). The text size was fixed (Ariel font, 60 point) and did not scale with screen size. Patients could pause or stop the therapy at any time. As long as the text was moving, a timer measured how much therapy was being delivered, feeding this information to the secure server. We suggested 20 min of therapy a day but patients could chose to do as much or as little as they wished. Patients were prompted to test their reading speeds after every 5 h of therapy had been accrued. Thus, the patients determined the time period between testing points.

### Subjective reports on reading behavior

At the end of the study all 33 patients were invited to provide more details, via a retrospective questionnaire. We asked the following: on which date did your visual field problem start? What caused your visual field problem? Has Read-Right helped your reading (rated from 1 [no benefit] to 10 [huge benefit])? And, compared to before starting Read-Right, every day I am reading for “X” amount of time more or less (rated from 7 options: the same amount; ±15; ±30; ±60 min) (see Table 1).

### Reading speed analysis

Reading speeds for each patient, at each 5-h time point, were entered into a repeated measures ANOVA to investigate the effects of therapy. In two sub-analyses we also added in the time taken to amass 5 and 10 h of therapy as a covariate; that is, we repeated the main analysis but with any explanatory effects due to this parameter removed. We assessed therapy effects after 5, 10, 15 and 20 h of therapy. The number of patients with data at each of the four points in time were: 33, 27, 20 and 18, respectively. Where the data violated sphericity assumptions, we report Greenhouse-Geisser corrected *p* and *F* values.

### Visual field analysis

For 7 subjects we did not obtain comparable visual field tests pre- and post-therapy. In four this was because they did not complete the second visual field test; for the other three we did not obtain tests of comparable sensitivity, as we improved the visual field test (made it more sensitive to visual loss) soon after launching the website. Points on the horizontal meridian in the right visual field were counted as missed or seen (4 = all four missed, 0 = all four seen) on both the pre- and post-therapy visual field tests and entered into a related-samples Wicoxon signed ranks test to assess significance.

## Results

**Fig. 2:**
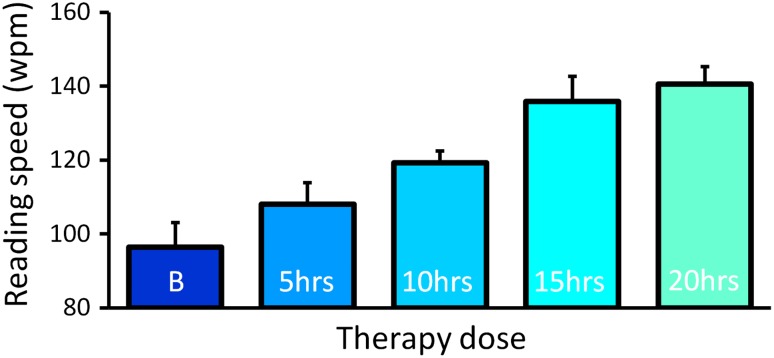
Cumulative effects of therapy in 5-h blocks. *Error bars* show the within-subject standard error of the means (6.6 at baseline (B); 5.7 at 5 h; 3.1 at 10 h; 6.9 at 15 h; 4.6 at 20 h). Age-matched controls’ reading speed have an average of 302 words/min with a SD of 80

There was a significant effect of Read-Right therapy at all four points in time. The size of effect increased monotonically, appearing to plateau at 20 h (Fig. 2). Effect sizes were as follows: 5 h 10.4 %, *F*(1, 32) = 8.68, *p* = 0.006; 10 h, 19.6 %, *F*(2, 25) = 6.34, *p* = 0.008; 15 h, 39.3 %, *F*(3, 17) = 7.21, *p* = 0.007; 20 h, 45.9 %, *F*(4, 14) = 8.91, *p* = 0.003.

Two alternative explanations are that this effect may be due to either practice effects (familiarity with the text reading test stimuli) or time effects (patients getting better as time passes). Fortunately, we were able to investigate these two possibilities because patients varied in how long it took them to reach each five hourly dose mark. We carried out two sub-analyses for the first two points in time (5 and 10 h therapy). Firstly, we calculated the correlation coefficient between percentage improvement and time to amass the therapy dose, and found no significant relationship: 5 h Pearson’s *r*(33) = −0.31 *p* = 0.08; 10 h Pearson’s *r*(26) = 0.06 *p* = 0.78. Secondly we added in the time to amass the therapy dose as a covariate into the ANOVAs as described above. Both remained significant: 5 h *F*(1, 31) = 12.44, *p* = 0.001; 10 h *F*(2, 24) = 3.25, *p* = 0.05.

A more prosaic explanation would be that the patients’ visual fields improved over time. However, we found no significant change in right hemifield vision: Wicoxon signed ranks test *Z* = −1.71, *p* = 0.09.

In response to reviewers’ comments, we carried out two post hoc analyses to investigate whether there was a correlation between the following: (a) the extent of the visual field defect and percentage improvement at 5 and 10 h; (b) patients’ ages and percentage improvement at 5 and 10 h. We used Pearson r correlation coefficient to test this. None of the analyses were significant: (a) visual field defect and % improvement at 5 h *r*(31) = 0.18, *p* = 0.31; and, at 10 h *r*(25) = −0.05, *p* = 0.79; (b) age and % improvement at 5 h *r*(31) = 0.23, *p* = 0.20; and at 10 h *r*(25) = −0.04, *p* = 0.84.

The subjective reports of improved reading behavior must be treated with caution as only 54 % of patients responded to the post hoc questionnaire and the chance of selection bias is high. Those who responded rated the Read-Right therapy as personally beneficial (mean 7.7/10), and 15/18 said they were reading more (median and mode “one hour or more a day”).

## Discussion

The principal finding of this study is that a clinically proven behavioral therapy can be delivered effectively to suitable patients using the internet. The effect sizes are in keeping with previous trials using this technique (range 23–113 %) [4, 9, 12]. In common with other behavioral therapies for language disorders, a dose effect was demonstrated [1], with post hoc analyses suggesting that this was not due to either practice effects on the testing materials or to the simple passage of time. There was a fair amount of individual variability with 27 % of patients showing no improvement after 5 h of therapy and 18 % not responding after 10 h (Table 1). There was no significant change in the patients’ visual fields.

While the patients’ subjective reports of improvement are encouraging, they need to be treated with caution because only 54 % responded to the post hoc questionnaire. This data was collected by asking the patients at the end of the study to respond. We did not collect the data prospectively because we did not want to over-burden patients with questions before they started treatment. In hindsight, this was a mistake as it introduces bias; we now ask all new patients to complete the questionnaire prior to starting the therapy.

Regarding patients with left-sided hemianopic alexia, there are some using the Read-Right web app, but less than with right hemianopias as patients with left-sided defects are less disabled [12]. We are planning a sub-analysis when we have enough subjects. One might expect such patients to improve with text scrolling in the other direction (to reverse the direction of the OKN induced saccade), but there is evidence that left-sided patients also benefit from leftward scrolling text [12].

If behavioral rehabilitation after acquired brain injury is viewed as a form of learning or re-learning, then the main lesson from the literature is that mass practice of a specific task is key in order to improve performance [2]; however, access to trained therapists is frustratingly limited for the majority of patients in the chronic phase post brain injury. Given this, and the fact that therapists’ time is more limited than patients’ capacity to improve [6], carefully designed, web-based resources like Read-Right represent a realistic way of delivering a large enough dose of therapy to patients for them to obtain clinically meaningful improvements.
